# Supplementary material for: FastqPuri: high-performance preprocessing of RNA-seq data
Source: BMC Bioinformatics. 2019 May 3;20:226. doi: 10.1186/s12859-019-2799-0 (PMC6500068; doi:10.1186/s12859-019-2799-0)
Supplement: Supplementary file 2 — Archive of FastqPuri. Archive containing all files needed to install and run FastqPuri v1.0.6. Date stamp March 22, 2019. (GZ 47,819 kb) [file 12859_2019_2799_MOESM2_ESM.gz › FastqPuri-1.0.6/html/md_README_trimFilter.html]

My Project: trimFilter user manual


My Project

trimFilter user manual

This program reads a `fastq` file as an input, and filters it according to the following criteria:

- Discard/trims reads containing adapter remnants.
- Discards reads matching contaminations (sequences collected in a `fasta` file or in an `idx` file created by `makeTree`, `makeBloom`
- Discards/trims low quality reads.
- Discards/trims reads containing N base callings.

## Running the program

Usage `C` executable (in folder `bin`):

Usage: trimFilter --ifq <INPUT\_FILE.fq> --length <READ\_LENGTH>

q --output [O\_PREFIX] --gzip [y|n]

--adapter [<ADAPTERS.fa>:<mismatches>:<score>]

--method [TREE|BLOOM]

(--idx [<INDEX\_FILE>:<score>:<lmer\_len>] |

--ifa [<INPUT.fa>:<score>:[lmer\_len]])

--trimQ [NO|ALL|ENDS|FRAC|ENDSFRAC|GLOBAL]

--minL [MINL] --minQ [MINQ]

(--percent [percent] | --global [n1:n2])

--trimN [NO|ALL|ENDS|STRIP]

Reads in a fq file (gz, bz2, z formats also accepted) and removes:

\* low quality reads,

\* reads containing N base callings,

\* reads representing contaminations, belonging to sequences in INPUT.fa

Outputs 4 [O\_PREFIX]\_fq.gz files containing: "good" reads, discarded

low Q reads discarded reads containing N's, discarded contaminations.

Options:

-v, --version prints package version.

-h, --help prints help dialog.

-f, --ifq fastq input file [\*fq|\*fq.gz|\*fq.bz2], mandatory option.

-l, --length read length: length of the reads, mandatory option.

-o, --output output prefix (with path), optional (default ./out).

-z, --gzip gzips output files: yes or no (default yes).

-A, --adapter adapter input three fields separated by colons:

<ADAPTERS.fa>: fasta file containing adapters,

<mismatches>: maximum mismatch count allowed,

<score>: score threshold for the aligner.

-x, --idx index input file. To be included with any method. 3 fields

3 fields separated by colons:

<INDEX\_FILE>: output of makeTree, makeBloom,

<score>: score threshold to accept a match [0,1],

[lmer\_len]: correspond to the length of the lmers to be

looked for in the reads [1,READ\_LENGTH].

-a, --ifa fasta input file. To be included only with method TREE

(it excludes the option --idx). Otherwise, an

index file has to be precomputed and given as parameter

(see option --idx). 3 fields separated by colons:

<INPUT.fa>: fasta input file [\*fa|\*fa.gz|\*fa.bz2],

<score>: score threshold to accept a match [0,1],

<lmer\_len>: depth of the tree: [1,READ\_LENGTH]. It will

correspond to the length of the lmers to be

looked for in the reads.

-C, --method method used to look for contaminations:

TREE: uses a 4-ary tree. Index file optional,

BLOOM: uses a bloom filter. Index file mandatory.

-Q, --trimQ NO: does nothing to low quality reads (default),

ALL: removes all reads containing at least one low

quality nucleotide.

ENDS: trims the ends of the read if their quality is

below the threshold -q,

FRAC: discards a read if the fraction of bases whose

quality lies below

the threshold -q is over 5 percent or a user

defined percentage in -p.

ENDSFRAC: trims the ends and then discards the read if

there are more low quality nucleotides than the

allowed by the option -p.

GLOBAL: removes n1 cycles on the left and n2 on the

right, specified in -g.

All reads are discarded if they are shorter than MINL.

-m, --minL minimum length allowed for a read before it is discarded

(default 25).

-q, --minQ minimum quality allowed (int), optional (default 27).

-p, --percent percentage of low quality bases to be admitted before

discarding a read (default 5),

-g, --global required option if --trimQ GLOBAL is passed. Two int,

n1:n2, have to be passed specifying the number of cycles

to be globally cut from the left and right, respectively.

-N, --trimN NO: does nothing to reads containing N's,

ALL: removes all reads containing N's,

ENDS: trims ends of reads with N's,

STRIPS: looks for the largest substring with no N's.

All reads are discarded if they are shorter than minL.

NOTE: the parameters -l or –length are meant to identify the length of the reads in the input data. Actually, `trimFilter` also copes with data holding reads with different lengths. The length parameter must hold the length of the longest read in the dataset.

## Output description

- `O_PREFIX_good.fq.gz`: contains reads that passed all filters (maybe trimmed).
- `O_PREFIX_adap.fq.gz`: contains reads discarded due to the presence of adapters.
- `O_PREFIX_cont.fq.gz`: contains contamination reads.
- `O_PREFIX_lowQ.fq.gz`: contains reads discarded due to low quality issues.
- `O_PREFIX_NNNN.fq.gz`: contains reads discarded due to *N*'s issues.
- `O_PREFIX_summary.bin`: binary file where information about the filtering process is stored. Structure of the file.
  - filters, `4*sizeof(int) Bytes`: array of int with entries `i = {ADAP(0), CONT(1), LOWQ(2), NNNN(3)}`. A given entry takes the value of the filter it was applied to and 0 otherwise. `filters[ADAPT] = {0,1}`, `filters[CONT] = {NO(0), TREE(1), BLOOM(2)}`,   
    `filters[LOWQ] = {NO(0), ALL(1), ENDS(2), FRAC(3), ENDSFRAC(4), GLOBAL(5)}`, `filters[trimN] = {NO(0), ALL(1), ENDS(2), STRIPS(2)}`.
  - trimmed, `4*sizeof(int) Bytes`: array of integers with entries i = {ADAP(0), CONT(1), LOWQ(2), NNNN(3)}, containing how many reads were trimmed due to the corresponding filter.
  - discarded, `4*sizeof(int) Bytes`: array of integers with entries i = {ADAP(0), CONT(1), LOWQ(2), NNNN(3)}, containing how many reads were discarded due to the corresponding filter.
  - good, `sizeof(int) Bytes`: number of accepted reads (maybe trimmed).
  - nreads, `sizeof(int) Bytes`: total number of reads.

## Filters

#### Adapters

Technical sequences within the reads are detected if the option `--adapters <ADAPTERS.fa>:<mismatches>:<score>` is given. The adapter(s) sequence(s) are read from the fasta file, and the search is done using an 'seed and extend' approach. It starts by looking for 16-nucleotides long seeds, for which a user defined number of mismatches is allowed (`mismatches`). If found, a score is computed. If the score is larger than the user defined threshold (`score`) and the number of matched nucleotides exceeds 12, then the read is trimmed if the remaining part is longer than `MINL` (user defined) and discarded otherwise. If no 16-nucleotides long seeds are found, we proceed with 8-nucleotides long seeds and apply the same criteria to trim/discard a read. A list of possible situations follows, to illustrate how it works (`MINL=25`, `mismatches=2`):

ADAPTER: GGCATACGAGCTCTTCCGATCT

REV\_COM: AGATCGGAAGAGCTCGTATGCC

CASE1A: CACAGTCGATCAGCGAGCAGGCATTCATGCTGAGATCGGAAGAGATCGTATG

||||||||||||X|||----

AGATCGGAAGAGCTCGTATG

- Seed: 16 Nucleotides

- Return: trimmed, TRIMA:0:31

CASE1B: CACATCATCGCTAGCTATCGATCGATCGATGCTATGCAAGATCGGAAGAGCT

||||||||------

AGATCGGAAGAGCT

- Seed: 8 Nucleotides

- Return: trimmed, TRIMA:0:37

CASE1C: CACATCATCGCTAGCTATCGATCGATCGATGCTATGCACGAAGATCGGAAGA

||||||||---

AGATCGGAAGA

- Seed: 8 Nucleotides

- Return: nothing done, reason: Match length < 12

CASE2A: CATACATCACGAGCTAGCTAGAGATCGGAAGAGCTCGTATGCCCAGCATCGA

||||||||||||||||------

AGATCGGAAGAGCTCGTATGCC

- Seed: 16 Nucleotides

- Return: discarded, reason: remaining read too short.

CASE2B: CCACAGTACAATACATCACGAGCTAGCTAGAGATCGGAAGAGCTCGTATGCC

||||||||||||||||||||||

AGATCGGAAGAGCTCGTATGCC

- Seed: 16 Nucleotides

- Return: trimmed, TRIMA:0:28

CASE3A: TATGCCGTCTTCTGCTTGCAGTGCATGCTGATGCATGCTGCATGCTAGCTGC

||||||||||||||||--

TATGCCGTCTTCTGCTTG

- Seed: 16 Nucleotides

- Return: discarded, reason: remaining read too short

CASE3B: CGTCTTCTGCTTGCCGATCGATGCTAGCTACGATCGTCGAGCTAGCTACGTG

||||||||-----

CGTCTTCTGCTTG

- Seed: 8 Nucleotides

- Return: discarded, reason: remaining read too short

CASE3C: TCTTCTGCTTGCCGATCGATGCTAGCTACGATCGTCGAGCTAGCTACGTGCG

||||||||---

TCTTCTGCTTG

- Seed: 8 Nucleotides

- Return: nothing done, reason: Match length < 12

The score is calculated as follows:

- - matching bases: `score += log_10(4)`
- - unmatching bases: `score -= Q/10`, where Q is the quality score.

#### Impurities

Contaminations are removed if a fasta or an index file are given as an input. Three methods have been implemented to check for contaminations:

- **TREE**: this method is designed to identify impurities from small sequences, such as rRNA, or EColi (not larger than 10MB). When using this method, ont has to pass one of these two options:
  - `--ifa <INPUT.fa>:<score>:<lmer_len>`: the file `INPUT.fa` is read and a tree of depth `lmer_len` is constructed on the flight.
  - `--idx <INDEX_FILE>:<score>`: in `INDEX_FILE` the tree structure was stored with `./makeTree`. A read will be considered to be a contamination if the score is bigger than `score`. The score is computed as the proportion of Lmers of a given read found in the tree. This method is very fast, every search is `O( 2 * Lmer * (L - Lmer + 1))` (the reverse complement has to be checked also), but has the drawback that is very memory intensive. This is why we have limited the construction of a tree to sequences whose size does not exceed 10 MB. Constructing a tree is very fast, that is why most of the times it might be not worth it to create the index file and then read it for every sample. Nevertheless, both options are provided.
- **BLOOM**: this method is designed for large sequences up to 4GB. An index is precomputed, using `makeBloom`, so that it is computed only once for all samples. The index file name and the threshold score have to be specified through the following option:
  - `--idx <INDEX_FILE>:<score>` The score is computed as for the previous options.

#### LowQ

- `--trimQ NO` or flag absent: nothing is done to the reads with low quality.
- `--trimQ ALL`: all reads containing at least one low quality nucleotide are redirected to `*_lowq.fq.gz`
- `--trimQ ENDS`: look for low quality (below MINQ) base callings at the beginning and at the end of the read. Trim them at both ends until the quality is above the threshold. Keep the read in `*_good.fq.gz` and annotate in the fourth line where the read has been trimmed (starting to count from 0) if the length of the remaining part at least `MINL`. Redirect the read to `*_lowq.fq.gz` otherwise. Examples (-q 27 [<] -m 25):

  ORIGINAL FILTERED

  @ read 1081133 @ read 1081133

  GTACATTGATGAGCAACATGGGGCTTGAACTGGCGCTGAAACAGTTAGGA GTACATTGATGAGCAACATGGGGCTTGAACTGGCGCTGAAACAGTTAGG

  +position: -144740 +position: -144740 TRIMQ:0:48

  IIIIIIIIIIIII9IIIIIIIIIIIIIIIIIIIIIIIIIIIIIIIIIII9 IIIIIIIIIIIII9IIIIIIIIIIIIIIIIIIIIIIIIIIIIIIIIIII

  @ read 3435 @ read 3435

  CAGTTCTTTGGTGTAGATGGAGCAGGACGGGATACCCATACCGTGACCCA CTTTGGTGTAGATGGAGCAGGACGGGATACCCATACCGTG

  +position: -19377 +position: -19377 TRIMQ:5:44

  99999IIIIIIIIIIIIIIIIIIIIIIIIIIIIIIIIIIIIIIII99999 IIIIIIIIIIIIIIIIIIIIIIIIIIIIIIIIIIIIIIII

  @ read 108110 @ read 108110

  CAGATAAATGCGAATGAATGGGTAACACTGGAGCTTTTAATGGCTGTAAC AGATAAATGCGAATGAATGGGTAACACTGGAGCTTTTAATGGCTGTAA

  +position: -4142524 +position: -4142524 TRIMQ:1:48

  9IIIIIIIIIIIIIIIIIII9IIII9III9IIIIIIIIIIIIIIIIIII9 IIIIIIIIIIIIIIIIIII9IIII9III9IIIIIIIIIIIIIIIIIII

  @ read 108111

  AGCGTGACGGTGTAACGCCCGCCTTTTGATGACTGGGTTTCAAAGAAACG

  +position: -3336785

  999999999999999IIIIIIII9IIIIIIIII9II99999999999999
- `--trimQ FRAC [--percent p]`: redirect the read to `*_lowq.fq.gz` if there are more than `p%` nucleotides whose quality lies below the threshold. `p=5` per default. Examples (-q 27 [<] -m 25 -p 5):

  ORIGINAL FILTERED

  @ read 1081133 @ read 1081133

  GTACATTGATGAGCAACATGGGGCTTGAACTGGCGCTGAAACAGTTAGGA GTACATTGATGAGCAACATGGGGCTTGAACTGGCGCTGAAACAGTTAGGA

  +position: -144740 +position: -144740

  IIIIIIIIIIIII9IIIIIIIIIIIIIIIIIIIIIIIIIIIIIIIIIII9 IIIIIIIIIIIII9IIIIIIIIIIIIIIIIIIIIIIIIIIIIIIIIIII9

  @ read 3435

  CAGTTCTTTGGTGTAGATGGAGCAGGACGGGATACCCATACCGTGACCCA

  +position: -19377

  99999IIIIIIIIIIIIIIIIIIIIIIIIIIIIIIIIIIIIIIII99999

  @ read 108110

  CAGATAAATGCGAATGAATGGGTAACACTGGAGCTTTTAATGGCTGTAAC

  +position: -4142524

  9IIIIIIIIIIIIIIIIIII9IIII9III9IIIIIIIIIIIIIIIIIII9

  @ read 108111

  AGCGTGACGGTGTAACGCCCGCCTTTTGATGACTGGGTTTCAAAGAAACG

  +position: -3336785

  999999999999999IIIIIIII9IIIIIIIII9II99999999999999
- `--trimQ ENDSFRAC --percent p`: first trim the ends as in the `ENDS` option. Accept the trimmed read if the number of low quality nucleotides does not exceed `p%` (default `p = 5`). Redirect the read to `*_lowq.fq.gz` otherwise. Examples (-q 27 [<] -m 25 -p 5): Missing reads would land in `*_lowq.fq.gz`.

  ORIGINAL FILTERED

  @ read 1081133 @ read 1081133

  GTACATTGATGAGCAACATGGGGCTTGAACTGGCGCTGAAACAGTTAGGA GTACATTGATGAGCAACATGGGGCTTGAACTGGCGCTGAAACAGTTAGG

  +position: -144740 +position: -144740 TRIMQ:0:48

  IIIIIIIIIIIII9IIIIIIIIIIIIIIIIIIIIIIIIIIIIIIIIIII9 IIIIIIIIIIIII9IIIIIIIIIIIIIIIIIIIIIIIIIIIIIIIIIII

  @ read 3435 @ read 3435

  CAGTTCTTTGGTGTAGATGGAGCAGGACGGGATACCCATACCGTGACCCA CTTTGGTGTAGATGGAGCAGGACGGGATACCCATACCGTG

  +position: -19377 +position: -19377 TRIMQ:5:44

  99999IIIIIIIIIIIIIIIIIIIIIIIIIIIIIIIIIIIIIIII99999 IIIIIIIIIIIIIIIIIIIIIIIIIIIIIIIIIIIIIIII

  @ read 108110

  CAGATAAATGCGAATGAATGGGTAACACTGGAGCTTTTAATGGCTGTAAC

  +position: -4142524

  9IIIIIIIIIIIIIIIIIII9IIII9III9IIIIIIIIIIIIIIIIIII9

  @ read 108111

  AGCGTGACGGTGTAACGCCCGCCTTTTGATGACTGGGTTTCAAAGAAACG

  +position: -3336785

  999999999999999IIIIIIII9IIIIIIIII9II99999999999999
- `--trimQ GLOBAL --global n1:n2`: cut all reads globally `n1` nucleotides from the left and `n2` from the right.

**Note:** qualities are evaluated assuming the reads to follow the L - Illumina 1.8+ Phred+33, convention, see Wikipedia. Adjust the values for a different convention.

#### N trimming

We allow for the following options:

- `--trimN NO` (or flag absent): Nothing is done to the reads containing N's.
- `--trimN ALL`: All reads containing at least one N are redirected to `*_NNNN.fq.gz`
- `--trimN ENDS`: N's are trimmed if found at the ends, left "as is" otherwise. If the trimmed read length is smaller than MINL, it is discarded. Example:

  ORIGINAL FILTERED

  @ read 1037 @ read 1037

  NNTCGACACAAGAAAATGCGCCAATTTTGAGCCAGACCCCAGTTACGCNN TCGACACAAGAAAATGCGCCAATTTTGAGCCAGACCCCAGTTACGC

  +position: -2044610 +position: -2044610 TRIMN:2:47

  IIIIIIIIIIIIIIIIIIIIIIIIIIIIIIIIIIIIIIIIIIIIIIIIII IIIIIIIIIIIIIIIIIIIIIIIIIIIIIIIIIIIIIIIIIIIIII

  @ read 1038

  AAAAAACGGTCGTGGTNGGCTACTGTTATTAAAGCGTTGGCTACAAAAAG

  +position: 1361068

  IIIIIIIIIIIIIIIIIIIIIIIIIIIIIIIIIIIIIIIIIIIIIIIIII

  @ read 1039

  CCAACGGACNATGGAAGTGCTNCGGCTGGNGTTTTTATCCTCCGGCATTC

  +position: -4282223

  IIIIIIIIIIIIIIIIIIIIIIIIIIIIIIIIIIIIIIIIIIIIIIIIII

  @ read 1043 @ read 1043

  AATCTGAAACGAAANCTGACAGCGNNCCCCGCTTCTGACAAAATAGGCGN AATCTGAAACGAAANCTGACAGCGNNCCCCGCTTCTGACAAAATAGGCG

  +position: -4685876 +position: -4685876 TRIMN:0:48

  IIIIIIIIIIIIIIIIIIIIIIIIIIIIIIIIIIIIIIIIIIIIIIIIII IIIIIIIIIIIIIIIIIIIIIIIIIIIIIIIIIIIIIIIIIIIIIIIII
- `--trimN STRIP`: Obtain the largest N free subsequence of the read. Accept it if is at least MINL nucleotides long, redirect it to `*_NNNN.fq.gz` otherwise. Example:

  ORIGINAL FILTERED

  @ read 1037 @ read 1037

  NNTCGACACAAGAAAATGCGCCAATTTTGAGCCAGACCCCAGTTACGCNN NNTCGACACAAGAAAATGCGCCAATTTTGAGCCAGACCCCAGTTACGCNN

  +position: -2044610 +position: -2044610 TRIMN:2:47

  IIIIIIIIIIIIIIIIIIIIIIIIIIIIIIIIIIIIIIIIIIIIIIIIII IIIIIIIIIIIIIIIIIIIIIIIIIIIIIIIIIIIIIIIIIIIIIIIIII

  @ read 1038 @ read 1038

  AAAAAACGGTCGTGGTNGGCTACTGTTATTAAAGCGTTGGCTACAAAAAG GGCTACTGTTATTAAAGCGTTGGCTACAAAAAG

  +position: 1361068 +position: 1361068 TRIMN:17:49

  IIIIIIIIIIIIIIIIIIIIIIIIIIIIIIIIIIIIIIIIIIIIIIIIII IIIIIIIIIIIIIIIIIIIIIIIIIIIIIIIII

  @ read 1039

  CCAACGGACNATGGAAGTGCTNCGGCTGGNGTTTTTATCCTCCGGCATTC

  +position: -4282223

  IIIIIIIIIIIIIIIIIIIIIIIIIIIIIIIIIIIIIIIIIIIIIIIIII

  @ read 1043

  AATCTGAAACGAAANCTGACAGCGNNCCCCGCTTCTGACAAAATAGGCGN

  +position: -4685876

  IIIIIIIIIIIIIIIIIIIIIIIIIIIIIIIIIIIIIIIIIIIIIIIIII

## Test/examples

The examples in folder `examples/trimFilter_SReport/` work in the following way:

1. See folder `examples/fa_fq_files`. The file `EColi_rRNA.fq` was created with `create_fq.sh` and contains:   
   \* 2e4 reads of length 50 from `EColi_genome.fa` with NO errors.
   - 5e3 reads of length 50 from `rRNA_modified.fa` with NO errrors (rRNA contaminations).   
     \* Artificially generated reads with low quality score (see `create_fq.sh`)
   - Artificially generated reads with Ns (see `create_fq.sh`).
   - Adapter files: `adapter_even_long.fa`, `adapter_odd_long.fa`, `adapter_even_short.fa`, `adapter_odd_short.fa`. Fasta files containing one adapter sequence each, longer/shorter than 16 nucleotides and with an even/odd length.
   - Example files to test the adapter contamination searchs: `human_[even/odd]_wad_[even/odd]_[long/short].fq`. Short fastq files where adapters contaminations have been inserted in all possible ways: even/odd positions, at the beginning/middle/end of the reads. Read lengths are even or odd as the first suffix indicates. The adapter contaminations included are suggested by the second even/odd suffix, and the long/short suffix.
2. `adapters/run_example.sh`: runs examples of reads containing adapters contaminations. A set of different possibilities is covered. See README file inside the folder `adapters`
3. `run_example_TREE.sh`: the code was tested with flags:

   $ ../../bin/trimFilter -l 50 --ifq PATH/TO/EColi\_rRNA.fq.gz

   --method TREE --ifa PATH/TO/rRNA\_modified.fa:0.9:50

   --trimQ ENDSFRAC --trimN STRIP -o tree

    i.e., we check for contaminations from rRNA, trim reads with lowQ at the ends and less than 5% in the remaining part, and strip reads containing N's. The output should coincide with the files `example_TREE*`   
   4. `run_example_BLOOM.sh`:   
   \* bloom filter is generated for `rRNA_modified.fa` with FPR = 0.0075 and `kmersize=25`. The output should coincide with `rRNA_example.bf*`.
   - trimFilter is run like in 2. but passing a bloom filter to look for contaminations with `score=0.4`.
4. With this set up, it is possible to run further customized tests.   
   **NOTE:** `rRNA_modified.fa` is the `rRNA_CRUnit.fa` sequence, where we have   
   removed the lines containing N's for testing purposes.

## Contributors

Paula Pérez Rubio

## License

GPL v3 (see LICENSE.txt)


---

Generated by  

 1.8.14
